# Inactivation of *Staphylococcus aureus* in water by pulsed spark discharge

**DOI:** 10.1038/s41598-017-10784-2

**Published:** 2017-09-04

**Authors:** Jiansheng Zheng

**Affiliations:** Oulin Central Research Institute, Oulin Group, Ningbo, Zhejiang Province 315000 China

## Abstract

A pulsed spark plasma discharge system was developed and tested as an energy efficient water sterilization method. A 5 log_10_ reduction on *Staphylococcus aureus* concentration of 10^8^ CFU/ml was obtained. Complete inactivation was achieved for concentration of 10^6^ CFU/ml. Of the various factors generated by an underwater spark discharge, ultraviolet radiation plays a major role. The inactivation was completely suppressed by the addition of 30 mg/L of a soluble sunscreen, Benzophenone-9. Results obtained using the pulsed spark plasma discharge showed that this system has several advantages, such as high energy efficiency, absence of harmful by-products and portability, over the conventional sterilization methods.

## Introduction

According to World Health Organization, nearly 1.1 billion people, which make up about 17% of the world’s population, don’t have access to clean and safe drinking water^1, 2^. Eighty percent of the illnesses in developing countries are caused by drinking unsafe water, killing 2–5 million people every year. Clean drinking water is essential for sustaining other critical societal needs such as education, economic development, nutrition and environmental health. In response to the increasing demand for water by world population, individual family size water collection and storage systems, including rooftop water tanks and rain harvesting systems, are being installed. However, water in these systems is stagnant, and thus may causes bacterial contamination and makes the water potentially unsafe to drink.

Conventional sterilization technology such as filtration, chlorination, UV radiation and ozonation have several drawbacks. Even though filters are effective in removing microorganisms, they are incapable of inactivating them. Hence, regular replacements of filters are required. The long term side effects of drinking chlorinated water are now being recognized^3^. Chlorine combines with natural compounds in water to form trihalomethanes, which trigger the production of free radicals in the body, causing tissue damage leading to cancer and reproductive defects^4–6^. Certain parasites like *Cryptosporidium* and *Giardia* are resistant to most sterilization methods like chlorination, and they can be easily transported through the distribution system to the point of use^7^. UV radiation is an effective tool for sterilizing water, but the total cost of UV water treatment, including energy cost, installation and maintenance, is relatively large^8^. Ozone is capable of inactivating microorganism in water comparable to chlorine, but at high temperatures and pH, ozone requires more exposure time due to its rapid decay^8^. Moreover, it is difficult to use these methods in developing countries with small, separated communities. Hence, there exists the need for a robust and environmental friendly water sterilization method for eliminate microorganisms in water, which can work effectively in small scales.

The use of plasma discharge in water for the sterilization of microorganisms in contaminated water proves to be a low cost and environmentally friendly technology^9–12^. Plasma discharges in water combine the contribution of strong UV radiation, high electric field, ozone, shock waves, and highly oxidative active species, to achieve the sterilization of contaminated water^8^. Generally, two main approaches are used to apply high voltage discharges for water sterilization: the first one is using high energy pulses, with the energy level of over 1 kJ per pulse^13^, and the second one is using low energy pulses, with the energy level of about 1 J per pulse^14^. The low energy pulse approach is especially attractive due to the simplicity in designing these systems into prototypes that can be integrated into household delivery systems such as well water units, rooftops etc. They can also be developed into a portable source of safe drinking water for military purposes and for foreign aid and disaster relief.

The high voltage pulsed discharge in water can be a partial discharge called the streamer-corona discharge or a fully developed spark or arc discharges. Two commonly used electrode geometries for generation of these discharges are the point-to-point geometry and point-to-plane geometry^15, 16^. The discharge starts usually from a sharp electrode. If the streamers do not reach the opposite electrode, it is referred to as corona discharge. A spark discharge is formed when these streamers reach the opposite electrode. If the current in the spark channel is above 1 kA, then it is called a pulsed arc discharge. Literature research and our preliminary results demonstrated that spark discharge is much more efficient in sterilization, and therefore our research was concentrated on studying spark discharge efficiency and mechanism.

In order to initiate a pulsed discharge directly in water, one needs to apply a very high intensity electric field on the order of 10^7^–10^9^ V/m at the tip of the electrode^10^. An appropriate insulation at the point electrode tip prevents energy losses due to electrolysis. The length of the point electrode extending beyond the insulation determines the stray electrolysis current and the discharge pattern. In the strong electric field region, microbubbles are formed by the local heating of water by the electrons^17^, though non-thermal mechanism of bubble formation may also be possible^18–20^. In the formed low density bubble, electron avalanches are created to form a discharge channel. The breakdown time, which is the time required for initiation of spark is dependent on the peak voltage. In the streamer regime of the discharge, the discharge current is limited by slow motion of ions from the streamer channel, while for spark discharge, the discharge channel connects two electrodes and the current is limited by the power supply system only. The temperature inside the spark discharge channel can reach over 10,000 K.

The factors that play an important role in inactivating microorganisms in water when a spark discharge is generated, are the generation of strong ultraviolet radiation, high intensity electrical field, free radicals, shock waves and metal nanoparticles produced as a result of electrode deterioration^21–23^. The energy is stored in plasma in the form of ionization, excitation, and kinetic energy of random particle movement. The stored energy is removed from plasma by electromagnetic radiation, shock waves and thermal conduction to the nearby water molecules, as the spark channel emits light, expands in size and the temperature inside the channel goes down^24^. Depending on the discharge type and total energy input, about thirty percent of the plasma energy can be radiated in the form of UV radiation^25^. The radical formation can be attributed to the following factors: electronic collision and UV photolysis. Some of the reactions that lead to the production of active species by electronic collision with the water molecules are^26^

1 $${H}_{2}O\to {H}+OH$$

2 $$2{H}_{2}O\to {H}_{2}{O}_{2}+{H}_{2}$$

The water surrounding the plasma absorbs the far UV generated by the spark discharge while near UV can cause photolytic decomposition of hydrogen peroxide produced by the discharge^11^

3 $${H}_{2}{O}_{2}+hv\to 2OH$$

The OH radicals produced by the pulsed discharge in water may recombine to form hydrogen peroxide^15^

4 $$OH+{OH}\to {H}_{2}{O}_{2}$$

with a reaction constant of 5 × 10^9^ M^−1^s^−1^.

Because of more intense excitation and ionization process, spark discharge is expected produce more hydrogen peroxide than corona discharge, given the same amount of energy input^10^.

Depending on the discharge energy, the plasma channel expansion can be sub- or supersonic. A transition from subsonic to supersonic expansion leads to the formation of shock waves and an increase in pressure inside the plasma channel^27^. The shock waves may interact with the microorganisms either causing them to explode or separate the colonies, thus exposing individual bacteria to UV and active chemical species^14^.

The current paper shows the effectiveness of the sterilization effect of the pulsed spark discharge in water contaminated with *Staphylococcus aureus* (*S. aureus*). The treatment system was optimized for obtaining higher energy efficiency with the spark discharge. The mechanism of inactivation was studied, and the results showed that ultraviolet radiation produced by the spark discharge plays a major role during the inactivation process of *S. aureus* in water.

## Results and Discussions

### Inactivation rate of S. aureus

**Figure 1:**
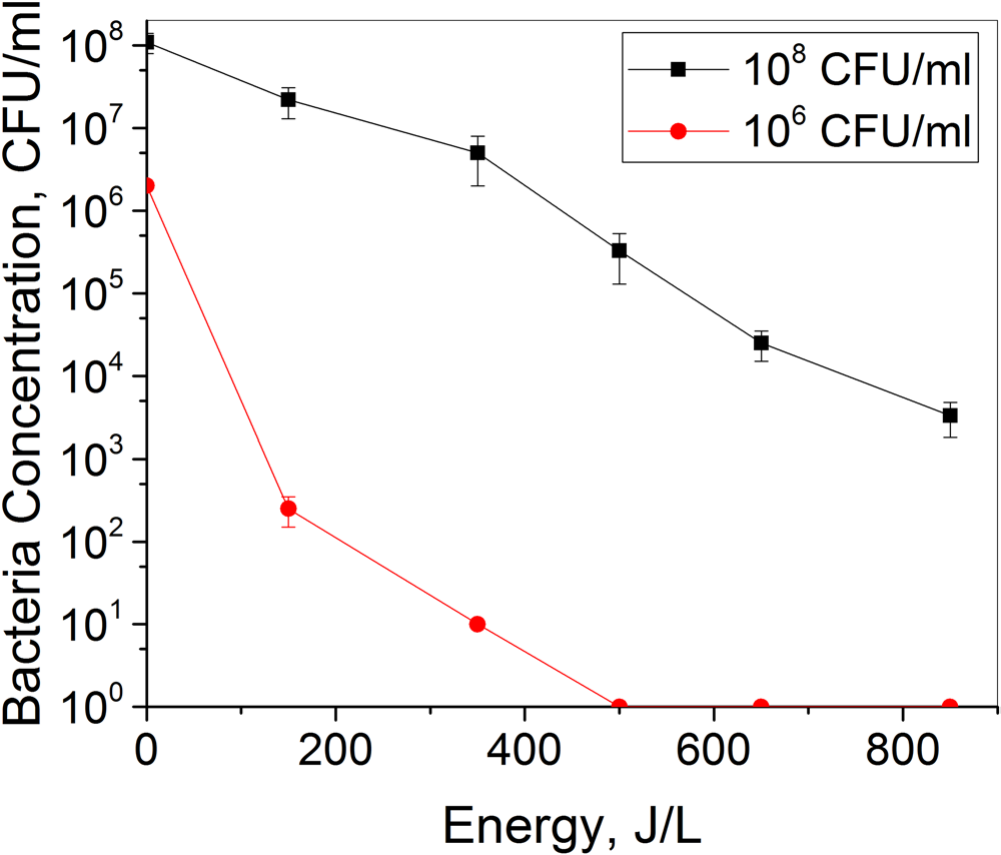
Sterilization rate for *S. aureus* concentration of 10^8^ and 10^6^ CFU/ml.

Experiments were performed for *S. aureus* concentrations of 10^6^ and 10^8^ CFU/ml, respectively. The purpose of using different concentrations was to check whether different D-value could be obtained for different bacterial concentrations. Figure 1 shows the inactivation rate obtained for the above concentrations.

For 10^8^ CFU/ml *S. aureus* concentration, the D-value was calculated to be 182 J/ L, which means that for 1 L of water, the energy cost to achieve 90% bacterial sterilization is 182 J. For 10^6^ CFU/ml *S. aureus* concentration, a D-value of 83 J/L was obtained, which was less than half of the value obtained for higher concentration. This indicates strong dependence of D-value on the bacterial concentration. The D-value increased with the increasing bacterial concentration. This effect may be explained by the fact that higher the bacterial concentration, murkier the water. As a result, the UV radiation generated by spark discharge was absorbed and unable to reach the bacteria. Another factor needs to be taken into consideration is that when the bacteria concentration is high, *S. aureus* tend to aggregate to each other. The aggregation can potentially shield the inner bacteria from the attacking of oxidation species, as well as the damage of shockwaves.

### Influence of UV radiation

To test the above hypothesis on the role of UV radiation, Benzophenone-9 (BP-9) was used to absorb the UV radiation in water. BP-9 is a sunscreen agent. It is non-toxic, and commonly used in commercial sunscreens^28–30^. By using different concentration of BP-9, it was shown that different degrees of UV absorption in water could be achieved. Fridman *et al*. demonstrated that for BP-9 solution with concentration of 3 mg/L, the water can still transmit a large part of UV radiation^31^. For BP-9 solution with concentration of 30 mg/L, significant part of UV radiation could be absorbed. For BP-9 solution with concentration of 3000 mg/L, the UV radiation could be completely absorbed. Hence in our current experiments, BP-9 solutions with concentrations of 3 mg/L, 30 mg/L and 3000 mg/L were prepared.

**Figure 2:**
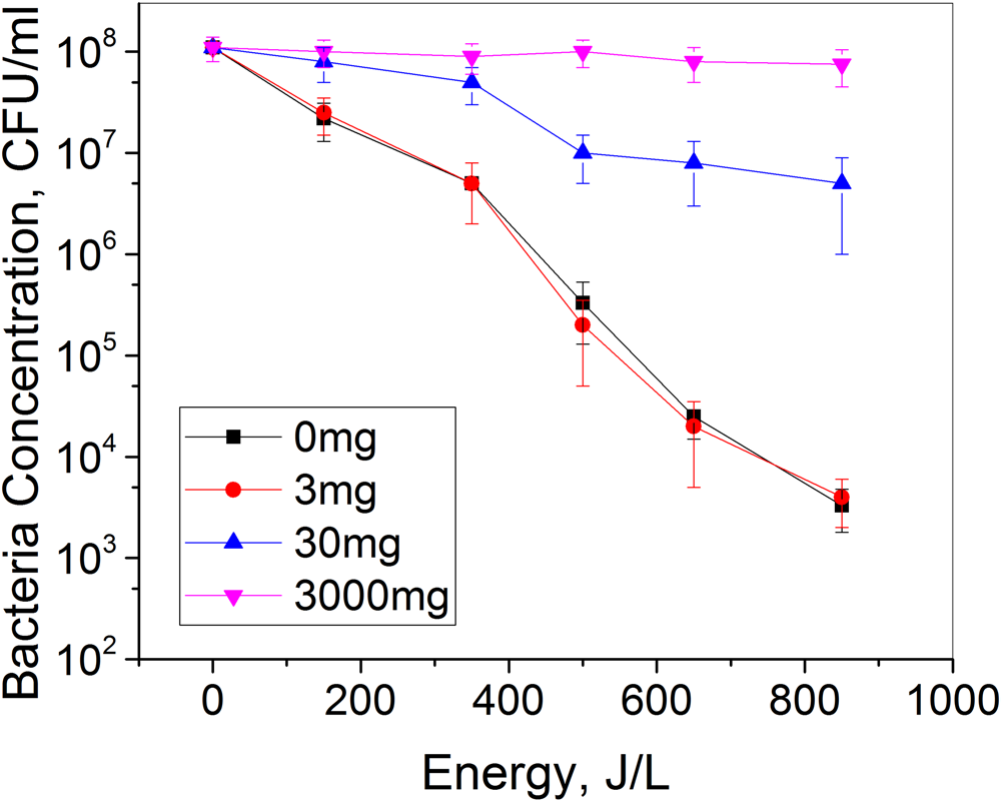
Addition of BP-9 influences the *S. aureus* inactivation efficiency of spark discharge plasma. Increasing concentrations (0–3000 mg/ml) of the soluble sunscreen BP-9 was added to water containing an initial *S. aureus* concentration of 10^8^ CFU/ml, treated with spark discharge plasma.

Figure 2 shows the inactivation rate of *S. aureus* for different concentrations of BP-9. It could be seen that for the case of 30 mg/L BP-9 in water, the disinfection of *S. aureus* in water was almost negligible. Similar experiments were also performed to study the role of UV radiation in the killing of water born microorganisms^32^. The results here proved that UV radiation plays a major role in the process of inactivating *S. aureus*.

BP-9 may have only minimal influence on reactive oxygen species (ROS) produced by plasma in water. Control experiments were done with BP-9 and H_2_O_2_ to check if the sunscreen scavenges H_2_O_2_. Measurements after 30 min following addition of 30 mg/L of BP-9 to 3 mg/L H_2_O_2_ resulted in no change in H_2_O_2_ concentration, indicating no scavenging action by BP-9. Also, BP-9’s benzyl rings is a very stable compound with a reaction constant at least 2 orders of magnitude less with respect to OH radicals. Hence, the presence of BP-9 may not change significantly the kinetics of OH recombination and formation of H_2_O_2_.

### Role of ROS in bacterial inactivation

**Figure 3:**
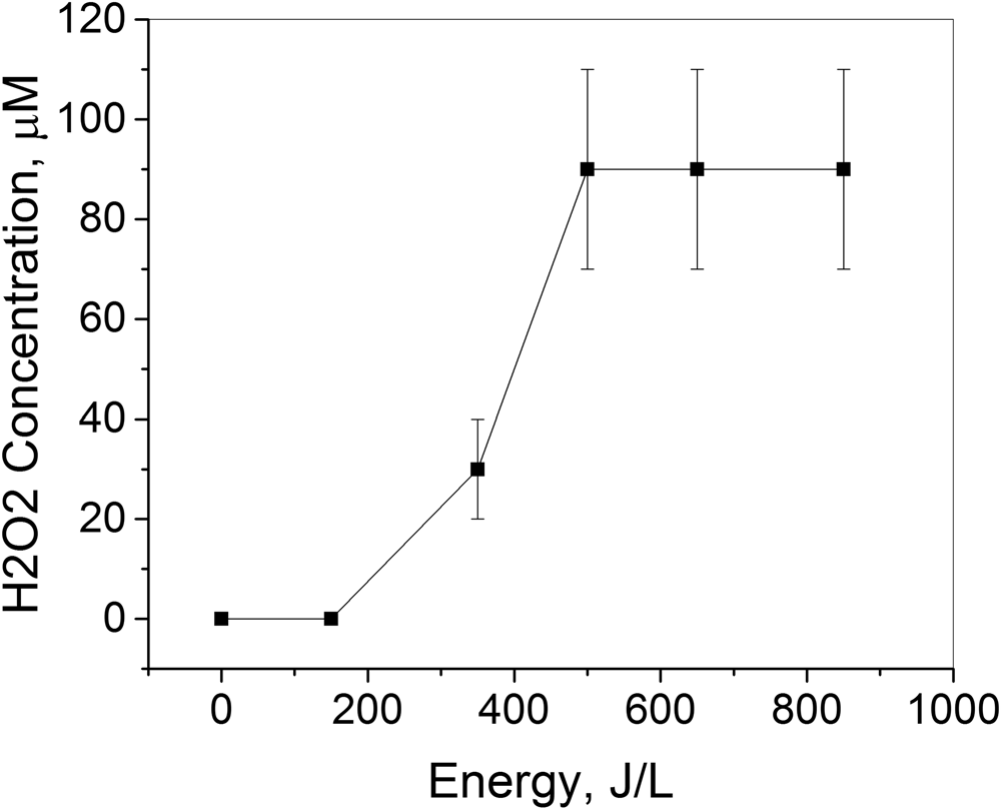
H_2_O_2_ produced by spark discharge plasma in water. Following plasma treatment, H_2_O_2_ in water was measured using peroxide test strips.

Hydrogen peroxide produced by spark discharge in spring water was measured for up to 850 J/L of energy input. An initial rise in H_2_O_2_ concentration was observed for up to 450 J/L followed by a saturation concentration of 90 μM up to 850 J/L (Fig. 3). Similar H_2_O_2_ saturation has been reported in pulsed corona treated water by Lukes *et al*., and was attributed to decrease in H_2_O_2_ yield due to H_2_O_2_ decomposition processes including UV photolysis (Equation 3) and decomposition by radicals (OH, HO_2_, H)^33^.

**Figure 4:**
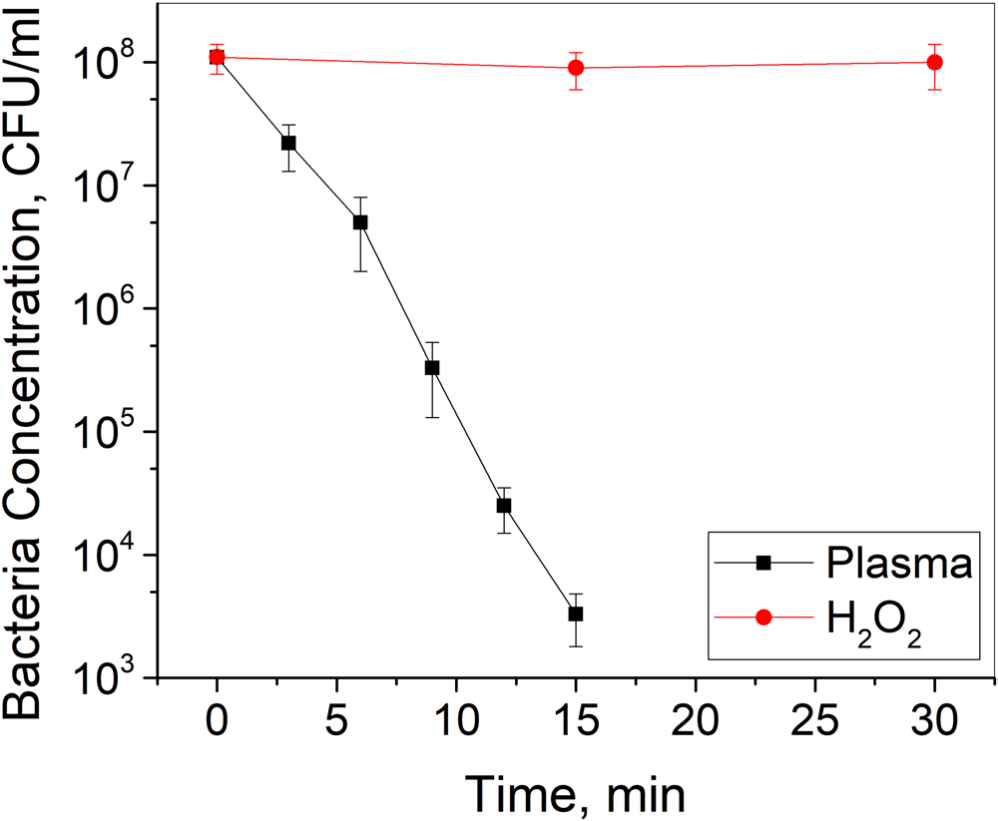
No change in bacterial concentration was observed with the addition of same concentration of H_2_O_2_ only. Water containing *S. aureus* concentration of 10^8^ CFU/ml was treated with spark discharge plasma or 90 μM of H_2_O_2_.

In order to evaluate the role of H_2_O_2_ in spark discharge plasma inactivation of *S. aureus* in water, 10^8^ CFU/ml of *S. aureus* in water was incubated with 90 μM of H_2_O_2_ for up to 30 min (Fig. 4). After 15 min of plasma treatment, which corresponds to an energy input of 850 J/L, a 5 log_10_ reduction in bacterial concentration was obtained. However, H_2_O_2_ treatment resulted in no change in bacterial concentration, indicating that H_2_O_2_ alone is not sufficient to produce S. aureus inactivation in our treatment system.

To further test the role of ROS in spark discharge inactivation of *S. aureus* in water, inactivation experiments were conducted in the presence of an ROS scavenger, N-acetyl cysteine (NAC, Sigma)^33^. Following incubation with 1 mM NAC, 10^8^ CFU/ml of *S. aureus* in water was subjected to plasma. Even in the presence of the ROS scavenger, a 5 log_10_ reduction in bacterial concentration was observed (Data not shown). Since NAC is a scavenger of multiple ROS such as OH and H_2_O_2_, these results indicate minimal influence of ROS in our spark plasma treatment system.

The OH radicals are generated mainly in spark discharge between electrodes, and immediately react with each other in the vicinity of spark channels with the formation of hydrogen peroxide in the reaction of recombination (Equation 4, k = 5 × 10^9^ M^−1^s^−1^). Fast recombination near the spark channels is one of the main reasons why OH is not the main component of bacteria inactivation in the volume.

### Optimization of the system

**Figure 5:**
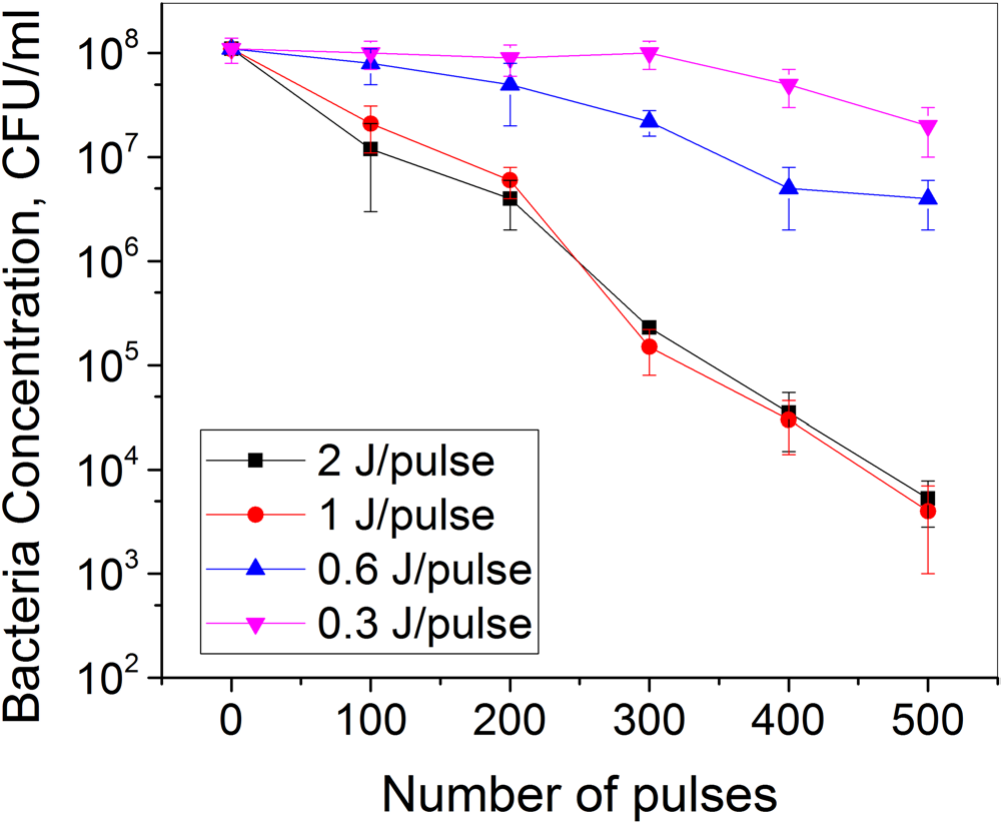
Survival plot obtained for *S. aureus* treated with different energies per pulse of spark discharge plasma. An initial *S. aureus* concentration of 108 CFU/ml was treated with different energies per pulse (0.3–2 J/pulse) of spark discharge plasma, and analyzed by plate count.

By changing the number of capacitor in the experiment setup, we can adjust the energy per spark pulse can deliver. Obtaining the dependence of D-value on the pulse energy would enable us to further optimize the setup. Figure 5 shows the inactivation rate of *S. aureus* for different energy levels. For energy level of 2 J/pulse, the D-value was 135 J/L. The lowest D-value was 90 J/L, corresponding to an energy level of 1 J/pulse. For energy levels low than 1 J/pulse, the D-values started to rise.

These results suggest optimal power range much smaller than that used by Ching *et al*.^32^. Additionally, a smaller discharge power would ensure longer electrode life.

Overall, spark treatment has several advantages over traditional water treatment methods as previously stated in Section 1. Filtration and chlorination treatments are not plasma generated which makes them difficult to directly compare but ozonation and UV are and should be compared to the spark discharge method. A general comparison of waste water treatment methods is presented since the concentration of bacteria and other contaminants plays a role in the treatment required as seen in Fig. 3. A review of industrial ozone generation methods showed an energy requirement of 10 kWh/kg to generate ozone from air^34^. The Neuilly-sur-Marne waste water treatment plant utilizes on an average 1.5 mg/L of ozone to disinfect contaminated water^35^. This suggests an energy requirement of 540 J/L for ozone water treatment compared to optimized value of 90 J/L for spark, indicating higher energy efficiency for spark. UV water treatment has been shown to be effective in bacteria reduction at 240 J/L^36^. Even though UV treatment is an established method of bacteria treatment in water, it has a limitation of biofouling of the lamp surface, which can reduce effective intensity. Therefore, some UV systems have rather complex methods to remove this fouling. Biofouling does not inhibit the discharge in plasma spark treatment due to the shockwave generated which would remove any potential biofilm growth. The spark system is comparable in energy use but is currently in a simplified state. With optimization of the configuration and hydrodynamics there is potential for a further increase of the efficiency and effectiveness.

## Methods

### Experimental setup

**Figure 6:**
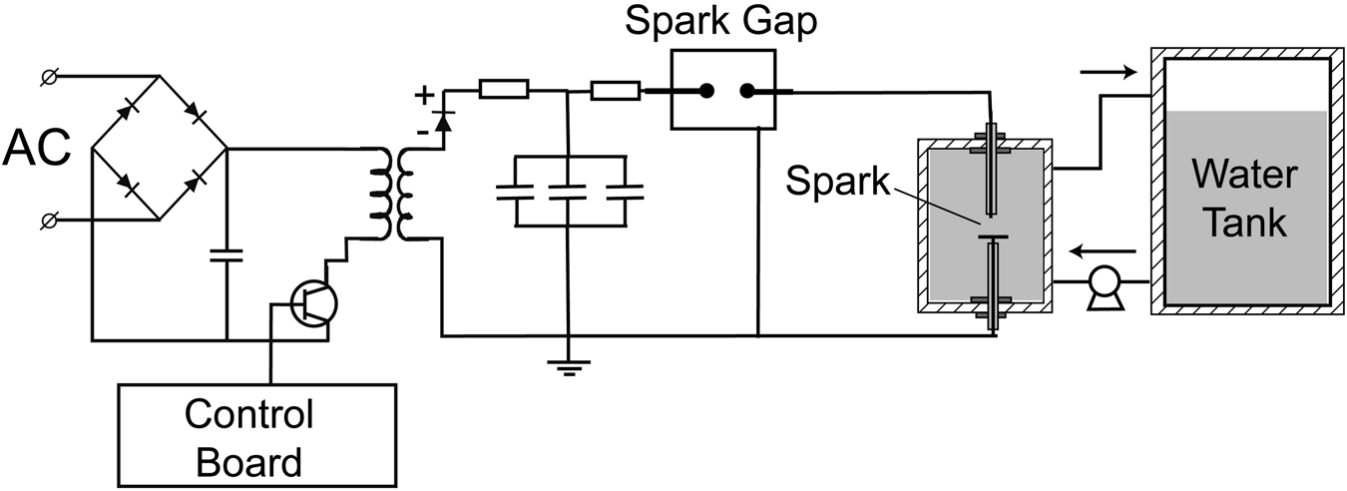
Schematic of the pulsed plasma discharge system. The experimental setup to treat water with spark discharge plasma comprises a power supply, capacitor bank, spark gap and point-to-plane electrode system.

The experimental setup consisted of a custom made high voltage power supply (Quinta LTD), a capacitor bank, a custom made spark gap and a point-to-plane electrode system. Figure 6 shows the schematic diagram of the optimized pulsed plasma discharge system and diagnostics.

The point-to-plane electrode system consisted of a stainless steel wire of diameter 0.2 mm, which is the anode, encased in silicone with Teflon tubing for electrical insulation purpose. To provide rigidity and dielectric strength to the electrode, it was placed in a plastic tubing. The stainless steel needle electrode extended approximately 1 mm beyond the bottom of the plastic tube. A grounded stainless steel plate, of diameter 120 mm, was used as the cathode. The gap between the two electrodes could be adjusted by moving the anode. Experiments were carried out with an electrode gap of 5 mm. The discharge chamber has a volume of 1 liter. The power supply charged the capacitor bank, of capacitance 9 nF, until the voltage was sufficiently high to initiate breakdown across the spark gap. The spark gap switch connected the capacitor bank to the high voltage electrode. The experiments were conducted with the following parameters: peak-to-peak voltage: 30 kV, peak-to-peak current: 100 A, pulse repetition frequency: 0.5 Hz, pulse duration: 1. 8 µs and energy per pulse: 0.3–2.0 J.

### Discharge characteristics

**Figure 7:**
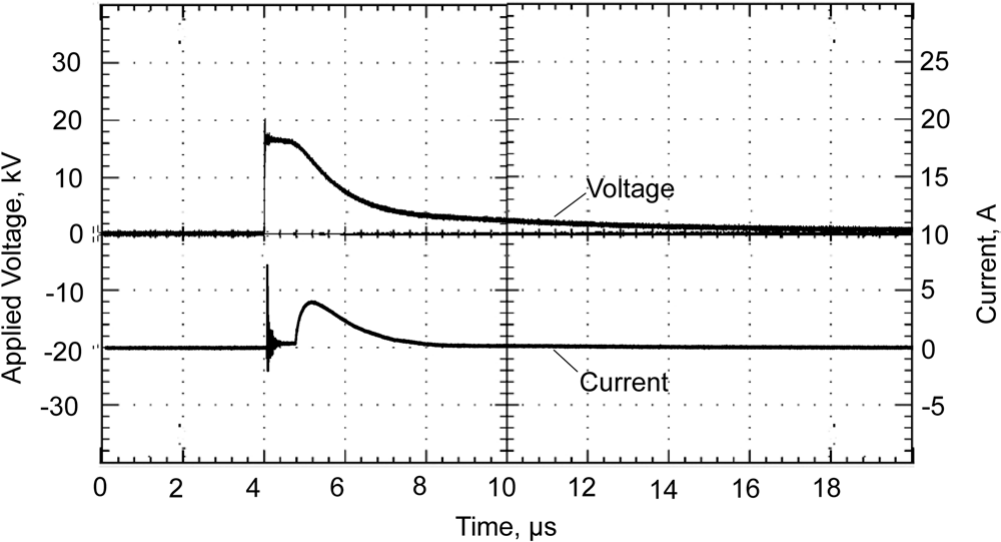
Typical waveforms of voltage and current obtained for a spark discharge in water.

A voltage probe 1:1000 (North Star PVM-4) and a current probe (Pearsons Model 2878) were used for measuring the voltage and current, respectively, to the high voltage electrode. The signals were recorded with an oscillograph (TDS 5052B, Tektronix Inc.) Fig. 7 shows the oscillograms of the voltage and the current during a spark discharge. Power and energy for spark discharge were calculated by numerical integration using MATLAB. Since a capacitor is the energy source, the current decreased after it reached a maximum value.

### Light emission spectrum

A monochromator (Princeton Instruments) was used to scan the emission spectra of the discharge. The spectra were obtained by a fiber optic cable. The cable was pointed at the discharge, with the other end connected to the monochromator. The response range of the monochromator was from 200 nm to 900 nm. An ICCD camera (Princeton Instruments) was triggered by the voltage signal from the voltage probe to record the emission.

**Figure 8:**
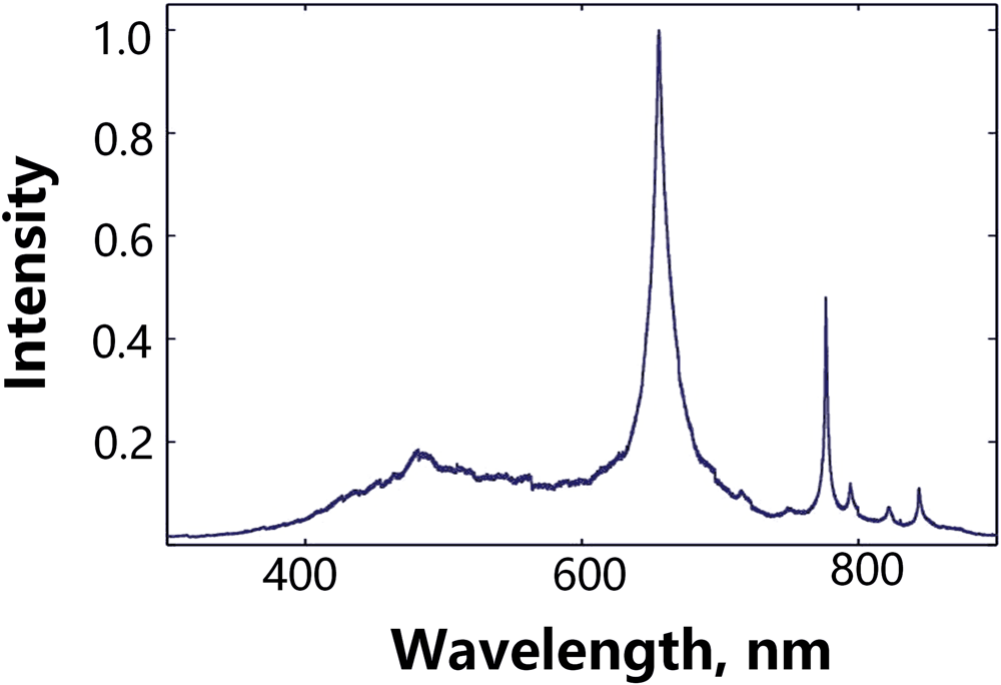
Underwater corona discharge optical emission spectra (300–900 nm).

Figure 8 shows the emission spectra of the underwater corona discharge. The signal was collected between the range of 300–900 nm, and integrated over the entire duration of the discharge. The spectra showed continuous radiation over the entire spectrum. In addition, atomic emission lines were observed due to the existence of hydrogen and oxygen radicals, possibly as a result due to the decomposition of water molecule. The most intense line is the H_α_ line at 656 nm. H_β_ line is visible at 484 nm, although very much broadened. Four lines due to the emission from atomic oxygen were present in the infrared region. Any possible radiation in the UVC range (from 200 nm to 300 nm) was buried under the continued decay of the H_β_ line. One thing worth noting is that no strong emission from metal atoms due to electrode erosion was visible in the spectra. For, the broadening could be caused by Stark broadening. The full width at half maximum (FWHM) of H_α_ line for corona discharge could be measured as 15 nm. This degree of broadening corresponds to an electron density of approximately 10^19^ cm^−3^ ^35, 37^. More broadening was observed for the H_β_ line. For the underwater spark, the broadening for the H_α_ line was about 35 nm, which corresponded to an estimated electron density of about 10^20^ cm^−3^.

### Microorganisms preparation

*S. aureus* is considered to be one of the most commonly used measure of public risks in food and drinking water^38^. A non-pathogenic strain of *S. aureus* was used in our current experiments. The bacteria were prepared by incubating cultures in the mixture of tryptone, yeast extract and sodium chloride for 24 hours at 37 °C^39^. They were then centrifuged at 3000 rpm for 5 minutes, washed in distilled water, and re-suspended in water to densities of 10^8^ to 10^6^ CFU/ml.

### Plasma treatment

Water with different density of *S. aureus* was treated with the plasma system and samples were taken at fixed time interval. The samples were diluted and prepared using sterile water. 100 µl of diluted water samples were spread on agar plates and incubated at 37 °C for 12 hours. The colony-forming units (CFU) were counted using a standard plate counting method to determine the bacterial concentration in water solution.

D-value is the energy required to achieve 90% (or one log_10_) reduction in bacterial concentration at the specific plasma treatment condition. This value is used to evaluate the efficiency of the spark discharge in sterilizing *S. aureus*.

### Hydrogen peroxide measurement

Hydrogen peroxide was measured in plasma treated water using EM Quant Peroxide test strips (VWR). Upon reacting with hydrogen peroxide, the test strip produces a blue oxidation product. Following plasma treatment (0–850 J/L), the test strip was dipped in plasma treated water for 1 sec, and excess water was removed using an absorbent towel. After 5 seconds, the peroxide concentration in treated water was measured semi-quantitatively by visual comparison of the reaction zone of the test strip with the fields of a color scale.
